# Inborn errors of metabolite repair

**DOI:** 10.1002/jimd.12187

**Published:** 2019-12-29

**Authors:** Maria Veiga‐da‐Cunha, Emile Van Schaftingen, Guido T. Bommer

**Affiliations:** ^1^ de Duve Institute Université Catholique de Louvain (UCLouvain) Brussels Belgium; ^2^ Walloon Excellence in Life Sciences and Biotechnology (WELBIO) UCLouvain Brussels Belgium

**Keywords:** 1,5‐anhydroglucitol‐6‐phosphate, D‐2‐hydroxyglutaric aciduria, enzyme promiscuity, G6PC3, G6PT, galactose, inborn errors of metabolism, metabolite repair, NADP(H)X, neutropenia, PGM1, SGLT2 inhibitor, UGP2

## Abstract

It is traditionally assumed that enzymes of intermediary metabolism are extremely specific and that this is sufficient to prevent the production of useless and/or toxic side‐products. Recent work indicates that this statement is not entirely correct. In reality, enzymes are not strictly specific, they often display weak side activities on intracellular metabolites (substrate promiscuity) that resemble their physiological substrate or slowly catalyse abnormal reactions on their physiological substrate (catalytic promiscuity). They thereby produce non‐classical metabolites that are not efficiently metabolised by conventional enzymes. In an increasing number of cases, metabolite repair enzymes are being discovered that serve to eliminate these non‐classical metabolites and prevent their accumulation. Metabolite repair enzymes also eliminate non‐classical metabolites that are formed through spontaneous (ie, not enzyme‐catalysed) reactions. Importantly, genetic deficiencies in several metabolite repair enzymes lead to ‘inborn errors of metabolite repair’, such as L‐2‐hydroxyglutaric aciduria, D‐2‐hydroxyglutaric aciduria, ‘ubiquitous glucose‐6‐phosphatase’ (G6PC3) deficiency, the neutropenia present in Glycogen Storage Disease type Ib or defects in the enzymes that repair the hydrated forms of NADH or NADPH. Metabolite repair defects may be difficult to identify as such, because the mutated enzymes are non‐classical enzymes that act on non‐classical metabolites, which in some cases accumulate only inside the cells, and at rather low, yet toxic, concentrations. It is therefore likely that many additional metabolite repair enzymes remain to be discovered and that many diseases of metabolite repair still await elucidation.

## 1 THE CLASSICAL VIEW OF METABOLISM

Work performed by the founders of intermediary metabolism led to the notion that enzymes are fantastic catalysts compared to those that are used by chemists. Enzymes are able to work under mild conditions of pH and temperature, they are often regulated and very importantly, they are remarkably specific. They act best on their physiological substrates, which are metabolites that make up the already known metabolic pathways. All this makes a lot of sense. Enzyme specificity was rightly assumed to prevent the production of substantial amounts of useless and potentially toxic side products. It allows also intermediary metabolism to be organised in metabolic pathways that involve a series of reactions, each catalysed by a ‘specific’ and dedicated enzyme. This organisation allows one to understand easily the major metabolic consequences of the enzyme defects causing inborn errors of metabolism. No doubt that this description of metabolism is largely true … but for the fact that enzyme specificity is not absolute!^1^, ^2^, ^3^

## 2 ENZYME IMPERFECTION

Articles describing new enzymes usually provide information on substrate specificity. In most of the cases, one compound is a much better substrate (usually 100‐ to 1000‐fold) than the other structurally similar molecules that have been tested. For instance, the glycolytic enzyme glyceraldehyde‐3‐phosphate dehydrogenase acts much better on glyceraldehyde‐3‐phosphate than on any other aldehyde that may occur in cells. Yet, in vitro, it also acts on erythrose‐4‐phosphate, an intermediate of the pentose phosphate pathway, at about 1/3000 of the rate of the ‘normal’ reaction.^4^, ^5^ Does this side reaction also occur in vivo? Yes, it does. The reason why we do not see any accumulation of 4‐phosphoerythronate in vivo is not that glyceraldehyde‐3‐phosphate dehydrogenase is more specific in its physiological context than in vitro, but simply that, as recently discovered,^5^ a ‘4‐phosphoerythronate phosphatase’ eliminates this side product in the intact cell (see below).

The study of inborn errors of metabolism makes us familiar with the idea that enzyme specificity is not perfect. The name commonly given to ‘phenylketonuria’^6^ (rather than hyperphenylalaninemia or phenylalanine hydroxylase deficiency) illustrates this very well. The accumulation of phenylpyruvate and phenylacetate in this disease is indeed most likely the consequence of side activities of transaminases and α‐keto acid dehydrogenases, stimulated by the elevated concentrations of phenylalanine. Numerous other examples exist of such promiscuous reactions taking place in the context of inborn errors of metabolism. If such reactions can be detected in vivo when the concentration of a promiscuous substrate is elevated due to a metabolic block, there is no reason to believe that they do not occur, at lower rates, under normal conditions.

The lack of absolute specificity of enzymes is the rule and not the exception.^3^ The description of enzymes with the lock‐and‐key model (though historically extremely important for the understanding of catalysis) is misleading in this respect. Proteins are flexible structures: the main polypeptide chain and the side chains of the amino acid residues may move to accommodate a molecule that is slightly different from the ideal substrate. This will of course result in less favourable kinetic properties, but the activity will still be significant.

## 3 THE INBORN ERROR OF METABOLISM L‐2‐HYDROXYGLUTARIC ACIDURIA ILLUSTRATES THE IMPORTANCE OF METABOLITE REPAIR

The study of L‐2‐hydroxyglutaric aciduria underlined the importance of metabolite repair.^7^ L‐2‐hydroxyglutaric aciduria results from a deficiency in L‐2‐hydroxyglutarate dehydrogenase, the FAD‐linked mitochondrial enzyme that catalyses the irreversible conversion of L‐2‐hydroxyglutarate to α‐ketoglutarate.^8^ L‐2‐hydroxyglutarate has no physiological role in higher organisms. It is made as a consequence of weak side‐activities of L‐malate dehydrogenase^9^, ^10^ and L‐lactate dehydrogenase^11^ that lead to the reduction of α‐ketoglutarate (structurally analogous to both oxaloacetate and pyruvate) to L‐2‐hydroxyglutarate. These side‐activities are extremely low, as illustrated by the observation that α‐ketoglutarate is more than one million times less good as a substrate for mammalian L‐malate dehydrogenase than oxaloacetate is.^10^ Yet, taking into account the enzyme abundance and the concentration of the substrates in tissues, we could calculate that L‐malate dehydrogenase catalyses the synthesis of several grams of L‐2‐hydroxyglutarate every day in a human adult.^8^ The same is true for lactate dehydrogenase. This considerable production of L‐2‐hydroxyglutarate, despite the high degree of specificity of L‐malate and L‐lactate dehydrogenase, implies that an additional mechanism is needed to prevent the accumulation of L‐2‐hydroxyglutarate. This is precisely the role of L‐2‐hydroxyglutarate dehydrogenase, a metabolite repair enzyme.^7^

If this metabolite repair enzyme is inactivated by mutations, L‐2‐hydroxyglutarate accumulates in tissues and particularly in the brain,^9^, ^12^ presumably because of the presence of the Na^+^‐dependent dicarboxylate transporter (NaDC3‐SLC13A3), which pumps dicarboxylic acids into astrocytes.^13^ Since L‐2‐hydroxyglutarate is a structural analog of α‐ketoglutarate, its accumulation in cells may inhibit several of the many enzymes that use this Krebs cycle intermediate as a substrate, as for instance histone demethylases, methylcytidine hydroxylase^14^, ^15^ and lysine‐α‐ketoglutarate reductase,^9^ the first enzyme of the lysine degradation pathway.

**Figure 1:**
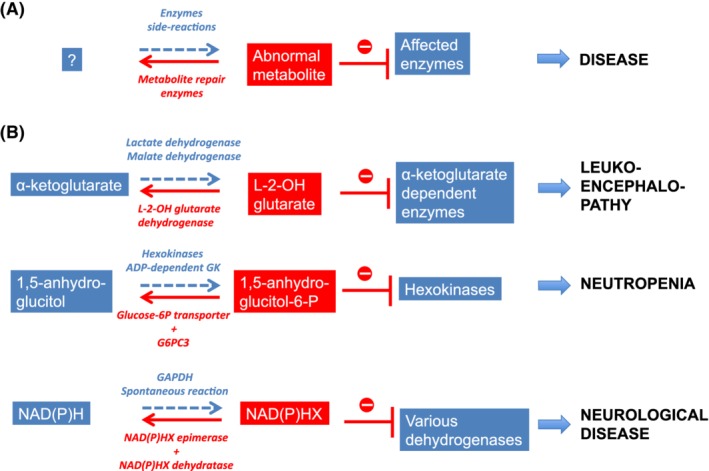
Metabolite repair and diseases of metabolite repair. Panel A, many enzymes catalyse side reactions leading to the production of abnormal metabolites. These metabolites may be toxic because they inhibit other enzymes. Fortunately, they are destroyed by metabolite repair enzymes and become unable to exert their toxic effects. Panel B, mutations inactivating the metabolite repair enzymes lead to various diseases such as L‐2‐hydroxyglutaric aciduria, to the neutropenia found in G6PC3 and G6PT deficiency and to the neurodegenerative disorders that are due to the deficiency in NAD(P)HX dehydratase or NAD(P)HX epimerase, as explained in the main text. Abbreviation: GAPDH, glyceraldehyde‐3‐phosphate dehydrogenase

The metabolite repair concept leads us to rephrase the statement on the importance of enzyme specificity in intermediary metabolism. ‘Enzymes of intermediary metabolism typically show a high degree of specificity. However, this specificity is not absolute and a special set of enzymes—metabolite repair enzymes—are needed to metabolize the side‐products resulting from the lack of absolute specificity’ (See Figure 1).^2^

## 4 A METABOLITE REPAIR DEFECT EXPLAINS THE NEUTROPENIA FOUND IN G6PC3 DEFICIENCY AND IN GLYCOGEN STORAGE DISEASE TYPE IB

Ubiquitous glucose‐6‐phosphatase (G6PC3) was discovered^16^, ^17^ as a homologue of glucose‐6‐phosphatase (G6PC1), the enzyme that produces glucose from glucose‐6‐phosphate in the endoplasmic reticulum of liver and kidney cells. Unlike G6PC1, G6PC3 is ubiquitously distributed in tissues and has very low glucose‐6‐phosphatase activity. In contrast to G6PC1 deficiency, which causes hypoglycemia, lactic acidosis and accumulation of glycogen in liver and in kidney (Glycogen Storage Disease type Ia), G6PC3 deficiency does not cause metabolic symptoms, but it is consistently associated with a severe neutropenia and, at least in humans, with heart, blood vessel and urogenital tract malformations.^18^, ^19^ Both G6PC1 and G6PC3 are trans‐membrane proteins that are inserted in the endoplasmic reticulum membrane with their catalytic site oriented towards the lumen of this organelle. A ubiquitously expressed glucose‐6‐phosphate transporter (G6PT, encoded by the SLC37A4 gene) mediates transport of glucose‐6‐phosphate from the cytosol to the lumen of the endoplasmic reticulum (reviewed in Reference 20). Its deficiency results in Glycogen Storage Disease type Ib,^21^, ^22^ which combines the same metabolic symptoms as G6PC1 deficiency with a severe neutropenia similar to that observed in G6PC3 deficiency.^23^

Neutrophils from patients with G6PT or G6PC3 deficiency were described as showing a reduced capacity to utilise glucose, decreased levels of ATP, a decreased respiratory burst, and a defect in protein glycosylation.^24^, ^25^ The mechanism by which a lack of G6PT or G6PC3 leads to neutropenia and neutrophil dysfunction remained an enigma until very recently, when we discovered that G6PC3 and G6PT collaborate to destroy a glucose‐6‐phosphate analog called 1,5‐anhydroglucitol‐6‐phosphate (best described as ‘1‐deoxyglucose‐6‐phosphate’).^26^ 1,5‐anhydroglucitol‐6‐phosphate is made from 1,5‐anhydroglucitol, a compound normally present in blood at 100 to 150 μM^27^ by side activities of glucose‐phosphorylating enzymes,^26^, ^28^ namely low Km hexokinases and ADP dependent glucokinase (ADPGK).^29^ 1,5‐anhydroglucitol‐6‐phosphate is a strong inhibitor of low Km hexokinases,^26^, ^30^ whose intracellular accumulation may therefore block glucose utilisation. What makes neutrophils particularly sensitive to hexokinase inhibition is the fact that the cells have lost virtually all their mitochondria during the maturation process. This makes them almost totally dependent on glucose metabolism.^31^, ^32^

**Figure 2:**
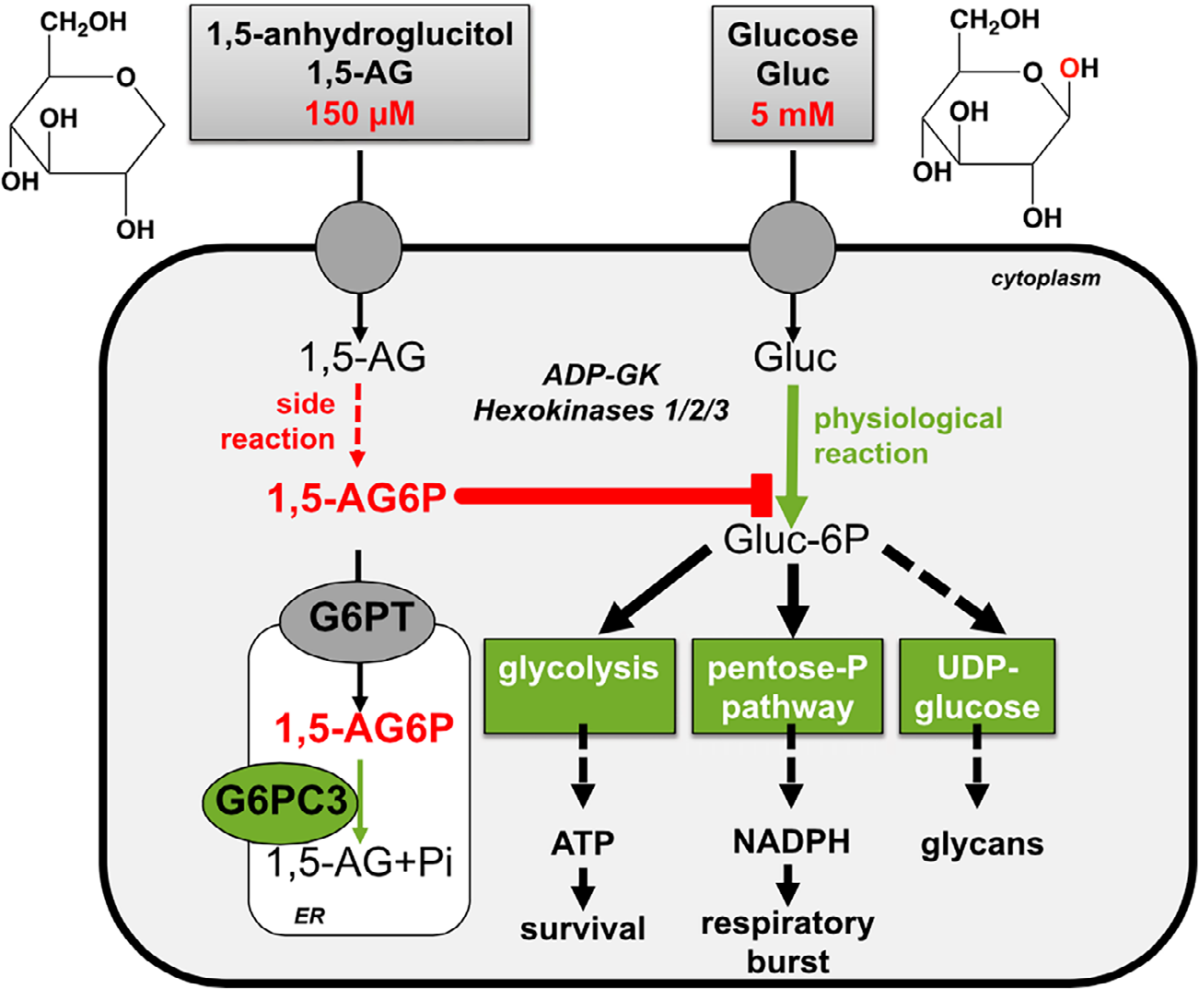
Role of G6PC3 and G6PT to maintain a low level of 1,5‐anhydroglucitol‐6‐phosphate and thereby prevent the toxic effects of this compound in neutrophils. 1,5‐anhydroglucitol, which is normally present in the blood at ≈ 100 μM, is phosphorylated to 1,5‐anhydroglucitol‐6‐phosphate by side activities of low‐Km hexokinases and of ADP dependent glucokinase (ADPGK). The glucose‐6‐phosphate transporter G6PT and G6PC3 collaborate to hydrolyse 1,5‐anhydroglucitol‐6‐phosphate, thereby preventing it to inhibit low Km hexokinases. This explains the lower glucose phosphorylation rates observed in neutrophils from patients with G6PC3 or G6PT transporter deficiency. G6PC3 was previously assumed to act as a glucose‐6‐phosphatase^64^ but as can be understood from this scheme, if this were the case, suppression of hydrolysis of glucose‐6‐phosphate should *increase* the flux through glycolysis and the pentose‐phosphate pathway, not decrease it (from Reference 26 with the required permission)

The proposed mechanism (Figure 2) is supported by many experimental data.^26^ Inactivation of G6PC3 or G6PT leads to marked accumulation of 1,5‐anhydroglucitol‐6‐phosphate in cells incubated with physiological concentrations of 1,5‐anhydroglucitol. Immortalised G6PC3‐deficient mouse neutrophil progenitors accumulate 1,5‐anhydroglucitol‐6‐phosphate and die when challenged with 1,5‐anhydroglucitol, while control cells are resistant to 1,5‐anhydroglucitol toxicity. The low number of neutrophils in G6PC3‐deficient mice can be normalised by lowering the concentration of 1,5‐anhydroglucitol in their blood with an inhibitor of the kidney sodium‐dependent glucose transporter (SGLT2).^33^ On the contrary, administration of 1,5‐anhydroglucitol to G6PC3‐deficient mice further decreases their neutrophil count. Neutrophils isolated from patients with G6PC3 or G6PT deficiency, show concentrations of 1,5‐anhydroglucitol‐6‐phosphate (≈ 3 mM) that are far above the Ki of hexokinase 3 (the main hexokinase in neutrophils)^34^ for this inhibitor, and close to 1000‐fold higher than the concentration found in neutrophils from healthy controls.^26^

Taken together, these findings indicate that G6PC3, in collaboration with G6PT, keeps the intracellular concentration of 1,5‐anhydroglucitol‐6‐phosphate low and thereby prevents hexokinase inhibition and a reduction in the rate of glucose metabolism in neutrophils. The neutropenia observed in G6PC3 or G6PT deficiency is therefore caused by a defect of metabolite repair (see Figure 1 **B**), which might be responsive to treatment with inhibitors of the Na^+^‐dependent glucose transporter of the kidney (SGLT2), currently used in the treatment of type II diabetes.

Most of the body pool of 1,5‐anhydroglucitol derives from food.^27^ Therefore, in principle, it could be interesting to propose a 1,5‐anhydroglucitol‐low diet to treat the neutropenia. However, designing such a diet may be difficult, because 1,5‐anhydroglucitol is present in most foods.^27^ Its origin is the breakdown of starch, glycogen or other glucosides to 1,5‐anhydrofructose, which is then enzymatically reduced to 1,5‐anhydroglucitol.^35^, ^36^ The degradation of glucosides to 1,5‐anhydrofructose is carried out by microbial enzymes known as alpha‐1,4‐glucan lyases, but it also happens as a weak side activity of some glucosidases, and this presumably explains the wide occurrence of 1,5‐anhydroglucitol and the fact that there is some endogenous production of this polyol in the human body. A recent genome‐wide association study of serum 1,5‐anhydroglucitol concentrations allowed the identification of seven loci, four of which were close to genes encoding enzymes involved in carbohydrate digestion in the gut.^37^ This suggests that the gut, and probably the bacteria that it contains, play an important role in the production of 1,5‐anhydroglucitol. This important point deserves further investigations.

G6PT has therefore three distinct functions: (a) to provide glucose‐6‐phosphate to G6PC1 in gluconeogenic tissues; (b) to provide glucose‐6‐phosphate to hexose‐6‐phosphate dehydrogenase,^38^ the NADPH‐producing enzyme of the endoplasmic reticulum; this reaction takes place in all tissues; (c) to transport 1,5‐anhydroglucitol‐6‐phosphate from the cytosol to the endoplasmic reticulum to allow its hydrolysis by G6PC3; the latter is a metabolite repair function that occurs in most tissues and cells, but is particularly critical for neutrophils. G6PT is therefore an example of a protein that participates both in classical metabolism and in metabolite repair.

Is the hydrolysis of 1,5‐anhydroglucitol‐6‐phosphate the only function of G6PC3? Possibly not, since G6PC3 has a wide substrate specificity,^26^ suggesting that it could have more than one physiological substrate. Therefore, it is possible that the lack of hydrolysis of one of these other substrates is at the origin of the malformations frequently observed in patients with mutations in G6PC3.

## 5 DEFICIENCY IN TWO SUPER‐CONSERVED REPAIR ENZYMES LEADS TO A NEW METABOLIC DISEASE

Coenzymes and cofactors have the appropriate reactivity to facilitate a whole diversity of reactions. However, because of their high reactivity, these molecules are more likely to be damaged than other more stable molecules. This is true for the pyridine nucleotides, NAD and NADP, which under their reduced form (NADH and NADPH), are rather easily converted to hydrated forms (called NADHX and NADPHX), that is, to forms that have undergone a water addition reaction. This reaction may for example be caused by a side activity of glyceraldehyde‐3‐phosphate dehydrogenase.^39^, ^40^ But it may also be spontaneous, and this is particularly true for NADPH, which is converted to NADPHX at a rate of 10% per hour at 37°C.^41^

The enzyme that reverses this damage by removing a water molecule from NAD(P)HX in an ATP‐dependent manner was first described in 1956.^42^ Yet, it was only recently that its sequence was identified.^41^ This raised the awareness that NADPHX dehydratase is an extremely conserved protein, which is present in virtually all eukaryotes, prokaryotes, and archaea. It also revealed the existence of an epimerase needed to change the orientation of the hydroxyl group resulting from the water addition reaction.^41^ As the dehydratase is specific for the *S*‐epimer of NAD(P)HX, the epimerase is required for the repair of the *R*‐epimer, which is equally formed when NADH or NADPH are hydrated. The epimerase is also an extremely conserved protein. This high conservation is not surprising: NAD and NADP are common to all living organisms and the problem of their hydration may have been particularly critical for the early forms of life, which appeared in surroundings with a high temperature. Indeed, increasing the temperature strongly enhances the rate at which spontaneous reactions take place. NADHX and NADPHX are known to inhibit several enzymes that use NAD or NADP as cofactors.^43^ This, together with the loss of functional nicotinamide nucleotides are certainly very good reasons to have a dedicated repair system that costs only one high energy bond per repaired NAD or NADP molecule (see Figure 1B).

Inactivating mutations in the epimerase^44^, ^45^ and in the dehydratase^46^ have recently been shown to cause a new form of leukoencephalopathy, which is characterised by the fact that fever episodes lead to a dramatic deterioration of the clinical state of the patients. In this disease, fever may not only result in a favoured unfolding of mutated enzymes, but also in enhancing the rate of formation of NAD(P)HX. The details of the pathophysiological mechanisms are still unknown, but both enzymes deficiencies lead to the accumulation of NADHX in cells and to perturbations in mitochondrial respiration.^47^

## 6 D‐2‐HYDROXYGLUTARIC ACIDURIA TYPE I, ANOTHER DEFECT OF METABOLITE REPAIR

**Figure 3:**
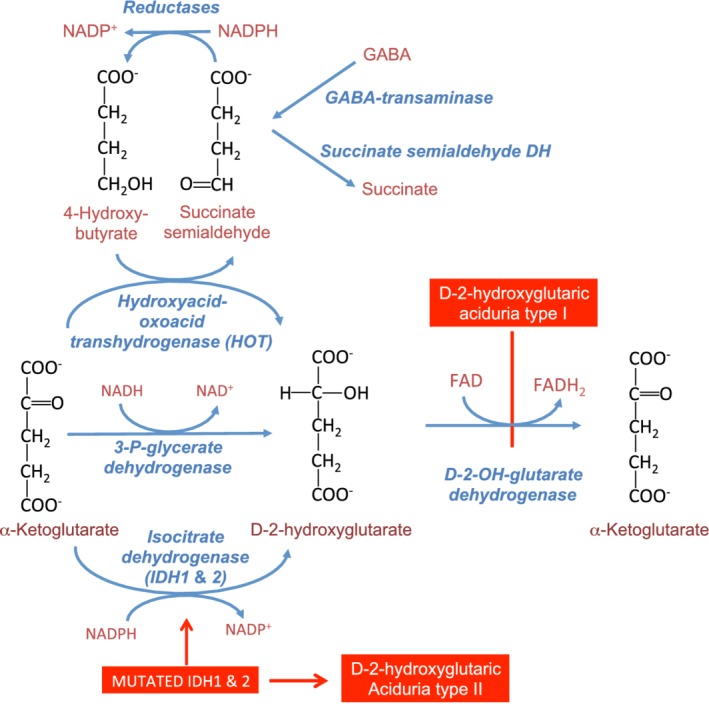
Production of D‐2‐hydroxyglutarate and its metabolism by the FAD‐dependent D‐2‐hydroxyglutarate dehydrogenase. The mitochondrial enzyme D‐2 hydroxyglutarate dehydrogenase catalyses the irreversible oxidation of D‐2‐hydroxyglutarate and its inactivation by mutations causes D‐2‐hydroxyglutaric aciduria type I. D‐2‐hydroxyglutarate can be produced from α‐ketoglutarate by four different enzymes. *Hydroxyacid‐oxoacid transhydrogenase* (*HOT*) oxidises 4‐hydroxybuyrate using alpha‐ketoglutarate as an electron acceptor. *3‐P‐glycerate dehydrogenase*, an enzyme involved in the pathway of serine synthesis (not shown), has a side activity on α‐ketoglutarate due to the structural similarity of the latter with 3‐phosphohydroxypyruvate, the normal product of this enzyme. Mutated forms of *IDH1* and *IDH2*, as described in various cancers, particularly in glioblastomas,^51^, ^52^ and in D‐2‐hydroxyglutaric aciduria type II (mutations in IDH2) very efficiently catalyse the reduction of α‐ketoglutarate to D‐2‐hydroxyglutarate; in this condition, the metabolic capacity of D‐2‐hydroxyglutarate dehydrogenase is exceeded and D‐2‐hydroxyglutarate accumulates

Two enzymes produce D‐2‐hydroxyglutarate from α‐ketoglutarate in normal mammalian cells (Figure 3). (a) 3‐Phosphoglycerate dehydrogenase, the first enzyme in the serine synthesis pathway shows a side activity on α‐ketoglutarate, a structural analog of the normal product of the 3‐phosphoglycerate dehydrogenase reaction, and reduces it to D‐2‐hydroxyglutarate^48^; (b) hydroxyacid: oxoacid transhydrogenase (HOT) an unusual oxidoreductase that uses α‐ketoglutarate as an electron acceptor, converting it to D‐2‐hydroxyglutarate. The physiological role of HOT is to oxidise 4‐hydroxybutyrate to succinate semialdehyde.^49^ This reaction is required because part of the succinate semialdehyde that is produced from GABA by GABA transaminase is transiently reduced by non‐specific aldehyde reductases to 4‐hydroxybutyrate, a metabolic dead end. Both sources of D‐2‐hydroxyglutarate are clearly the result of enzyme side activities, rather than mainstream metabolism.

D‐2‐hydroxyglutarate dehydrogenase is the repair enzyme that metabolises D‐2‐hydroxyglutarate. It has common features with L‐2‐hydroxyglutarate dehydrogenase, in that it is an FAD‐linked mitochondrial enzyme that irreversibly converts its substrate to α‐ketoglutarate.^50^ Its deficiency leads D‐2‐hydroxyglutaric aciduria type I, which because of the origin of its substrate, is also a metabolite repair defect. D‐2‐hydroxyglutarate dehydrogenase has a very high affinity for D‐2‐hydroxyglutarate (Km < 10 μM), but only a very low metabolic capacity, which is sufficient to cope with normal production of D‐2‐hydroxyglutarate, but can be overwhelmed when production increases.

In several cancer cells, and particularly glioblastoma, mutations of isocitrate dehydrogenase 1 or 2 (IDH1 and IDH2) make that these enzymes become NADPH‐dependent α‐ketoglutarate reductases and no longer display their normal isocitrate dehydrogenase activity.^51^, ^52^ Hereditarily transmitted mutations of IDH2 with a similar effect are found in patients with D‐2‐hydroxyglutaric aciduria type 2.^53^ Mutated IDH1 or IDH2 become so efficient at producing D‐2‐hydroxyglutarate that the low metabolic capacity of D‐2‐hydroxyglutarate dehydrogenase is no longer adequate to prevent D‐2‐hydroxyglutarate accumulation.

It is outside the scope of this short review to discuss the role of D‐2‐hydroxyglutarate in oncogenesis. Intriguingly, patients with D‐2‐hydroxyglutaric aciduria type I show widely variable clinical spectra, and the same is true for the type II form.^54^ This is possibly the result of D‐2‐hydroglutarate affecting the pattern of gene expression by inhibiting α‐ketoglutarate dependent enzymes such as proline hydroxylase, histone demethylases, and methylcytosine hydroxylase.^14^, ^15^

## 7 HOW MANY METABOLITE REPAIR ENZYMES ARE THERE?

The rapid pace of discovery of new metabolite repair enzymes in recent years^2^, ^55^ indicates that many metabolite repair enzymes still remain to be discovered. Glycolysis is the metabolic pathway for which the inventory of metabolite repair enzymes is the most complete.^55^ Eleven different repair reactions have been identified. In addition to those described above and encoded by the genes *L2HGDH*, *G6PC3*, *NAXD*, and *NAXE*, we should add *PGP* (encoding phosphoglycolate phosphatase), which destroys three toxic glycolytic side products: 4‐phosphoerythronate, L‐2‐phospholactate, and 2‐phosphoglycolate^5^; glyoxalase I and II, which destroy methylglyoxal, a toxic compound formed from triose‐phosphates spontaneously or by a side activity of triose‐phosphate isomerase^56^; and finally two enzymes that serve to repair glycation products resulting from the spontaneous reaction of glucose or glucose‐6‐phosphate with amines.^57^, ^58^ Thus, the number of known repair reactions just matches the number of classical enzymatic reactions involved in the conversion of glucose to lactate. If this ratio of about one repair enzyme per conventional enzyme applies to the whole metabolic map, we are probably still missing hundreds of metabolite repair enzymes, and possibly the explanation for many inborn errors of metabolism.

## 8 WHEN IS A METABOLITE REPAIR ENZYME CRITICAL?

**Figure 4:**
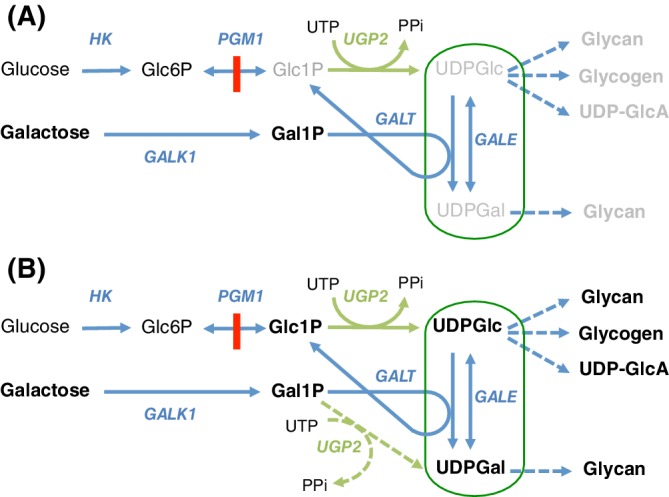
Enzyme promiscuity explains that galactose supplementation can correct glycosylation defects in PGM1 deficiency. UDP‐glucose pyrophosphorylase (UGP2), known to form UDP‐glucose (UDPGlc) from glucose‐1‐phosphate (Glc1P) and UTP, has a side activity (shown as dashed green arrows in panel B) that allows it to make UDP‐galactose (UDPGal) from UTP and galactose‐1‐phosphate (Gal1P). This side activity is essential to explain that galactose supplements correct the metabolic problems in PGM1 deficiency (illustrated by the low concentrations of the metabolites shown in grey in panel A). When PGM1 is deficient, cells do not have the possibility of making UDP‐glucose and UDP‐galactose from glucose because they cannot make glucose‐1‐phosphate from glucose‐6‐phosphate (Glc6P). Galactose supplementation raises the concentration of galactose‐1‐phosphate. Panel A, In the absence of the side activity of UGP2, galactose‐1‐phosphate cannot be converted to UDP‐galactose (in grey), since the co‐substrate of GALT, UDP‐glucose (in grey), is deficient. Panel B, However, when the side activity of UGP2 is taken into account (shown in green dashed arrows), the small production of UDP‐galactose from galactose‐1‐phosphate and UTP initiates a virtuous cycle that inflates the pool of UDP‐hexoses and explains the clinical benefits of galactose supplementation

Two main reasons justify the existence of a metabolite repair enzyme. The main one is that the repair enzyme removes a toxic metabolite. This accounts for the importance of eliminating NAD(P)HX, D‐2‐hydroxyglutarate, L‐2‐hydroxyglutarate and 1,5‐anhydroglucitol‐6‐phosphate (see Figure 1B) and 4‐phosphoerythronate.

Another reason may be to recover potentially useful metabolites. Accordingly, Nit1 which is a highly conserved amidase found in animals, yeast, plants, and many bacteria, hydrolyzes deaminated glutathione, a damaged form of glutathione resulting from the side activities of various transaminases.^59^ Nit1 thereby allows the cells to recycle the useful molecules of which deaminated glutathione is made of, that is, α‐ketoglutarate, cysteine and glycine. Interestingly, absence of this repair enzyme in mice, yeast, eukaryotic cell lines and *Arabidopsis thaliana* has no detrimental consequences, despite the striking accumulation (and urinary loss) of deaminated glutathione.^59^, ^60^ Deaminated glutathione is likely innocuous. Yet, because of the important urinary loss of deaminated glutathione induced by its accumulation, the deficiency of Nit1 may increase the need for sulfur amino acids in the diet and therefore be problematic when the diet is deficient in these.

Therefore, it is likely that diseases of metabolite repair occur mostly when the abnormal metabolite that needs to be destroyed is toxic, because, in most of the cases, it acts as an inhibitor of one or several enzymes of intermediary metabolism. In these cases when the abnormal metabolite is an extremely potent inhibitor (4‐phosphoerythronate^5^; NADHX^46^), low levels of accumulation are enough to cause dramatic perturbations. Identifying the toxic compound may be very difficult in the absence of ad hoc hypotheses, particularly if this compound is an unknown metabolite. Yet, identifying the exact function of a metabolite repair enzyme in the context of human inborn errors of metabolism may be very useful as it may lead to new therapeutic strategies, as appears to be the case for 1,5‐anhydroglucitol‐6‐phosphate.^26^

Many diseases of metabolite repair likely remain to be elucidated. This may be the case of diseases for which the mutated gene is already known, but for which the function of the encoded product is unknown. If the encoded protein is ‘enzyme‐like’ or even simply if it is conserved in very distant organisms like bacteria (metabolic enzymes are the most conserved proteins), the possibility that it is a yet unknown enzyme of metabolite repair needs to be taken into consideration, and the adequate strategy followed to understand its function.

## 9 EPILOGUE: WHEN ENZYME PROMISCUITY IS USEFUL

As often observed in bacteria, enzyme promiscuity may be advantageous in the context of enzyme defects, also in humans. A nice example is phosphoglucomutase 1 (PGM1) deficiency, which causes problems in the synthesis of glycans because of defective production of glucose‐1‐phosphate and therefore of UDP‐glucose (by UDP‐glucose‐pyrophosphorylase—UGP2) and UDP‐galactose (by UDP‐galactose epimerase—GALE) (Figure 4A). Remarkably, the glycosylation defect in this disease is efficiently treated by galactose supplements,^61^ which in fibroblasts and presumably also in vivo increase the UDP‐glucose and UDP‐galactose pools.^62^, ^63^ The mechanism of this effect is more complex than it would appear at first sight.

Galactose is phosphorylated to galactose‐1‐phosphate (GALK1), which is converted to UDP‐galactose by transfer of a uridylyl moiety from UDP‐glucose by uridylyltransferase (GALT). Importantly, this transuridylylation reaction *does no*t increase the (UDP‐glucose + UDP‐galactose) pool (circled in green in Figure 4), even if large amounts of galactose‐1‐phosphate are present, because one molecule of UDP‐glucose is consumed for each molecule of UDP‐galactose that is formed in the transuridylylation reaction. There is therefore no net formation of UDP‐hexose (Figure 4A).

The simplest way of explaining the increase in the UDP‐hexose pool under these conditions is to assume that galactose‐1‐phosphate can be converted to UDP‐galactose by a reaction that uses another uridylyl donor than UDP‐glucose. Such a reaction does indeed exist: specificity studies^63^ have shown that UDP‐glucose pyrophosphorylase (UGP2) has a weak (a few percentage of the classical reaction) but significant side activity, where UTP and galactose‐1‐phosphate are converted to UDP‐galactose and inorganic pyrophosphate (UTP + galactose‐1‐phosphate ➔ UDP‐galactose + PPi)^63^ (Figure 4B). This reaction is certainly facilitated by the high concentration of galactose‐1‐phosphate resulting from the galactose supplements, and by the low concentration of glucose‐1‐phosphate (one of the substrates of UGP2) due to the PGM1 deficiency. The UDP‐galactose that is formed in this way can be converted to UDP‐glucose by the epimerase (GALE) and, via the GALT reaction, replenish the glucose‐1‐phosphate pool. Glucose‐1‐phosphate is in turn converted to a second molecule of UDP‐glucose, initiating a virtuous cycle that progressively expands the UDP‐hexose pool and will cease only when galactose‐1‐phosphate is exhausted (Figure 4). The important point to stress here is the fact that this virtuous cycle is initiated by a useful side activity of UGP2.

To conclude, this *a contrario* example does not detract from the importance of metabolite repair. It just highlights the complexity of metabolism and how far we need to go in the details of pathophysiological mechanisms to provide the best help to the patients. There is little doubt that the number of diseases that will be explained by a default in metabolite repair mechanisms will greatly expand in the coming years.

### AKNOWLEDGMENTS

The work performed in the authors' laboratory is supported by the Fonds de la Recherche Scientifique‐FRS/FNRS (J.0104.18 to MVDC), Walloon Excellence in Life Sciences and Biotechnology (WELBIO CR‐2015A‐09 to EVS; WELBIO 2019‐PARKINSON to GB), the European Research Council (ERC) under the European Union's Horizon 2020 research and innovation programme grant agreement (No 771704 to GB), the Fédération Belge contre le Cancer (2016‐075 to GB), the de Duve Institute and UCLouvain. MVDC is ‘Chercheur qualifié’ of the FNRS. GB is ‘Maître de Recherche’ of the FNRS.

**Abbreviations**
D2HGDH: D‐2‐hydroxyglutarate dehydrogenase
G6PC3: ubiquitous glucose‐6‐phosphatase
G6PT: glucose‐6‐phosphate translocase of the endoplasmic reticulum
GALE: UDP‐galactose epimerase
GALK1: galactokinase
GALT: galactose‐1‐phosphate uridylyltransferase
HK: hexokinase
L2HGDH: L‐2‐hydroxyglutarate dehydrogenase
NAXE: NAD(P)HX dehydratase
NAXE: NAD(P)HX epimerase

## CONFLICT OF INTEREST

Maria Veiga‐da‐Cunha, Emile Van Schaftingen and Guido Bommer declare that they have no conflict of interest.

## AUTHOR CONTRIBUTIONS

All authors have contributed to the reporting of the work described in the article.
